# Relative Effects of Demographic, Psychological, Behavioral, and Social Factors on the Initiation and Maintenance of Leisure-time Physical Activity: Results From a Confirmatory Path Analysis in a Longitudinal Study

**DOI:** 10.2188/jea.JE20200073

**Published:** 2021-11-05

**Authors:** Jaesung Choi, JooYong Park, Ji-Eun Kim, Jong-koo Lee, Daehee Kang, Miyoung Lee, Ick-Joong Chung, Ji-Yeob Choi

**Affiliations:** 1 Institute of Health Policy and Management, Seoul National University Medical Research Center, Seoul, Korea; 2 BK21plus Biomedical Science Project, Seoul National University College of Medicine, Seoul, Korea; 3 Department of Biomedical Sciences, Seoul National University Graduate School, Seoul, Korea; 4 JW Lee Center for Global Medicine, Seoul National University College of Medicine, Seoul, Korea; 5 Department of Family Medicine, Seoul National University College of Medicine, Seoul, Korea; 6 Department of Preventive Medicine, Seoul National University College of Medicine, Seoul, Korea; 7 Cancer Research Institute, Seoul National University, Seoul, Korea; 8 Institute of Environmental Medicine, Seoul National University Medical Research Center, Seoul, Korea; 9 College of Physical Education and Sport Science, Kookmin University, Seoul, Korea; 10 Department of Social Welfare, Ewha Womans University, Seoul, South Korea

**Keywords:** determinants, physical activity, initiation, maintenance, path analysis

## Abstract

**Background:**

There is a lack of evidence of the complicated pathways of underlying determinants in the phases of physical activity. The purpose of this study was to evaluate simultaneously a set of potential determinants on the initiation and maintenance phases of leisure-time physical activity (LTPA).

**Methods:**

The longitudinal data of 54,359 Korean adults aged 40–69 years from the Health Examinees study were used. The median follow-up duration was 4.2 years. The self-reported durations per week of LTPA was repeatedly assessed. Based on previous longitudinal studies, the potential determinants were selected, and hypothetical models were constructed that consider the complex associations between the determinants. The standardized coefficients for direct and indirect effects were estimated using path analysis to differentiate contributions of mediation from the total effects.

**Results:**

In the total population, age, education, chronic diseases, smoking, depression symptoms, and self-rated health were significantly associated with both initiation and maintenance phases. Income (*B* = 0.025) and social supports (*B* = 0.019) were associated only with the initiation phase. Waist-to-hip ratio (*B* = −0.042) and stress (*B* = −0.035) were associated only with the maintenance phase. After stratifying by sex, the significant effects of education, chronic diseases, and smoking were found only in men. The initiation phase-specific effects of income and social supports and the maintenance phase-specific effects of stress were found only in women. It was estimated that indirect effects contributed approximately 15% of the total effect.

**Conclusion:**

The findings suggested that there were initiation- or maintenance-specific determinants of leisure-time physical activity according to sex.

## INTRODUCTION

Participation in regular physical activity is known to be associated with health benefits.^1^^–^^3^ According to global recommendations on physical activity for health,^4^ it is suggested that at least 150 minutes of moderate intensity or 75 minutes of vigorous intensity physical activity per week be performed. However, in recent decades, insufficient physical activity has become a global public health problem that has not improved.^5^ Thus, it is important to identify the factors underlying changes in physical activity, called determinants, and to encourage people to participate in physical activity.

A previous review of reviews summarized results finding that physical activity is influenced by several determinants in multi-faceted categories, including demographic, psychological, and environmental.^6^ However, those summarized results mainly came from individual analysis that can only assess the total effect of a single determinant; the complex causal pathway of a large set of determinants that can be used to assess direct and indirect effects simultaneously has not been considered. In addition, there were a relatively insufficient number of results on behavioral determinants. To evaluate the comprehensive effects of determinants, it is necessary to construct a hypothetical model that considers various determinants in multifaceted categories based on previous literature.

From the behavioral hypothetical stage model, it was known that distinct determinants affect behavior changes at different phases.^7^ For physical activity, two phases have been defined in previous systematic reviews^8^^,^^9^: initiation, a period in which people begin to be more physically active; and maintenance, a period of continuous participation in regular physical activity extending to habit development. They suggest that initiation-specific determinants are included in the psychological and social categories, but which fail to identify maintenance-specific determinants. This can result from differences in the causal path of the determinants between initiation and maintenance. For example, it has been suggested that behavioral initiation is related to favorable expectations regarding future outcomes, whereas behavioral maintenance is related to satisfaction with received outcomes.^10^ Thus, the phase-specific causal paths need to be evaluated.

The purpose of this study was to construct an a priori hypothetical model of potential determinants based on previous literature, and to evaluate the direct and indirect contributions of potential determinants to the initiation and maintenance of leisure-time physical activity (LTPA), using a longitudinal design.

## METHODS

### Study population

This longitudinal study was conducted in the Health Examinees-Gem (HEXA-G) study derived from the Health Examinees study, a component of the Korean Genome and Epidemiology Study (KoGES_HEXA). The characteristics of the cohort have been described elsewhere.^11^^,^^12^ Briefly, the KoGES_HEXA is a large-scale cohort study that recruited participants aged 40 to 69 between 2004 and 2013 (*N* = 173,345) from 38 health examination centers and hospitals across the eight regions of Korea. All baseline participants received an invitation to the follow-up conducted between 2012 and 2016. Among the participants of KoGES_HEXA, 139,344 participants were included in the HEXA-G study, after excluding participants who met the following criteria (eFigure 1): aged younger than 40 years or older than 69 years, because they were a non-target age cohort (*N* = 3,627); and recruited from centers that had only operated in a pilot study, had different processes for quality control and biospecimen collection, had been participating for fewer than 2 years, or were no longer participating in the cohort (*N* = 30,374). Of those participants, 64,485 were included in the follow-up (follow-up rate = 46.2%). The median follow-up duration was 4.2 years. We additionally excluded the participants who were recruited in the first year of the baseline (*N* = 1,697) and who had missing values in the determinants and outcome variables (*N* = 8,429). Finally, 54,359 participants were included in the analysis.

During recruitment, a consent form was voluntarily signed by all of the participants before entering the study. The Institutional Review Board of Seoul National University Hospital, Seoul, Korea approved the study (IRB No. 0608-018-179).

### Hypothetical model construct

**Figure 1.**
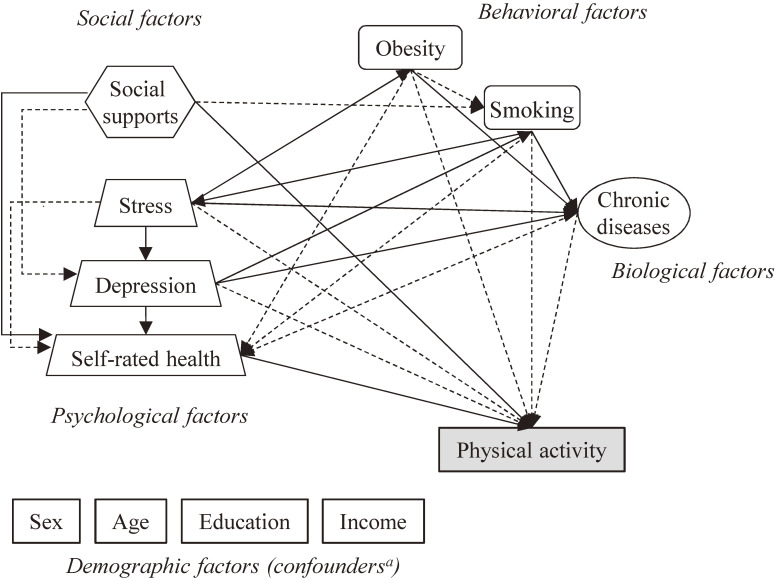
Hypothetical model developed based on previous literature. Solid and dash lines indicate the positive and negative associations, respectively. ^a^Demographic factors, such as sex, age, education, and income, were included in the model as confounders and linked to all other variables

We previously summarized a list of 117 factors that have been reported for their effects on physical activity by the review of reviews.^6^ Among those factors, 20 available factors were selected from HEXA-G: age, sex, marital status, education, income, occupational type, employment, grip strength as indicator of fitness, chronic diseases, injury history, stress, self-rated health, quality of life, depression symptoms, dietary habits, obesity, alcohol, smoking, screening, and social supports. To confirm the associations of these factors with physical activity, a two-stage review of previously literature^6^^,^^13^ was performed. First, we searched associations between each factor and physical activity reported in a meta-analysis, as used in Lampure et al.^13^ Second, the associations of each factor with physical activity were defined if there were more than four prospective results and 50% of the results supported the expected association, as used in Lampure et al^13^ and Choi et al.^6^ Finally, sex, age, education, income, social support, stress, depressive symptoms, obesity, smoking, chronic disease, and self-rated health were selected as potential determinants of LTPA (eTable 1). We also reviewed associations between the potential determinants, using the same methods described earlier. Finally, the model was constructed as in Figure 1; solid and dashed lines indicate the positive and negative associations, respectively. Sex, age, education, and income were included in the hypothetical model as confounders, because it is known that these factors are associated with other determinants of physical activity.

### Assessment of potential determinants

Potential determinants collected through a self-reported questionnaire at baseline were used as continuous or ordinal categorical variables in the analysis. In the demographic and biological category, age (continuous), educational level (≤middle school, high school graduate, and ≥college), monthly income (<2.0 million, 2.0–3.9 million, and ≥4.0 million won [1 million won is approximately US $1,000]), and chronic diseases were assessed. Chronic diseases were assessed as the number of past diagnoses of chronic diseases, including hypertension, diabetes, cardiovascular diseases (CVDs), and cancer (ranging from 0 to 4). The list of chronic diseases was selected based on common diseases for which effects on physical activity have been reported.^14^^–^^24^ In the psychological, cognitive, and emotional categories, stress (not at all, often, and frequent), self-rated health (poor, normal, and good), and depression symptoms were assessed. Depression symptoms were assessed through a shortened form of the psychosocial well-being index (PWI_SF) corrected for the General Health Questionnaire-60.^25^ Among the 18 items of a four-point Likert scale, we used the total score of three items assessing depression symptoms (score ranged 0–9). In the social and cultural category, social support was assessed by the score of received emotional support, including having someone to confide in and provide emotional care (0–2). Social capital was estimated by summing the z-score of the following four variables: the number and contacting frequency of close family members and the number and contacting frequency of close relatives, friends, colleagues, and neighbors. In behavioral factors, smoking (nonsmoker for 10 years and current smoker) and obesity-related factors were assessed. As an indicator of obesity, waist-to-hip ratio (WHR; the ratio of the circumference of the waist to that of the hips) and body mass index (BMI; weight in kilograms divided by height in meters squared) were assessed as continuous variables.

### Assessment of leisure-time physical activity

Self-reported information on physical activity was collected at both baseline and follow-up. At both periods, participants were asked whether they participated in regular LTPA enough to sweat. Those who participated in LTPA were then asked, “How many times do you participate in regular exercise enough to sweat per week?” and “On average, how many minutes did you regular exercise per session?” The LTPA duration per week was calculated by multiplying the duration of each session by the frequency per week. This variable was used as the overall duration of LTPA. Participants were then classified based on the recommendations from the international guidelines for physical activity^4^: non-participating, <150 minutes per week, and participating, ≥150 minutes per week. Changes in duration of LTPA between baseline and follow-up were calculated by comparing the categories of the duration of LTPA (eTable 2). There were three levels of change in LTPA (0, +1, and +2) among those who did not participate in LTPA at baseline, and there were three levels (−2, −1, and 0) among those who participated in 150 minutes per week or more of LTPA. Negative and positive levels of changes in LTPA indicated a decrease or increase of adherence to LTPA, respectively.

The level of physical activity collected using the questionnaire was evaluated for validity and reliability by comparing the results with the objectively measured physical activity from an accelerometer (ActiGraph, Pensacola, FL, USA), a multi-sensor (SenseWear Armband monitors, BodyMedia Inc., Pittsburgh, PA, USA), and global physical activity questionnaire (GPAQ).^26^ In brief, LTPA from HEXA was positively correlated with MET-hour per day from ActiGraph (rho = 0.343, *P* < 0.05), average energy expenditure/day from the multi-sensor (rho = 0.272, *P* < 0.05), and level of LTPA from the GPAQ (rho = 0.222, *P* < 0.05). The level of physical activity obtained from the HEXA also had moderate-to-high reliability in repeated measurements at 3-month test–retest intervals (interclass correlation coefficient, ICC = 0.722).

### Statistical analysis

To compare the associations between each determinant and the overall LTPA from our study to the summarized results of previous studies (eTable 1), a generalized linear model (GLM) was used to evaluate the sex and age-adjusted least square means (lsmeans) and beta estimates of the overall LTPA duration at follow-up per unit or category of potential determinants at baseline, according to the level of LTPA at baseline (non-participated and participated in 150 minutes or more physical activity per week). The distributions of social support, social capital, and duration of LTPA, which had either a skewness greater than 3.0 or kurtosis greater than 10.0, were transformed using the Box–Cox method.^27^ The *P*-values for trend were evaluated for ordinal variables by calculating the linear association with the duration of the LTPA by a GLM. Interactions of each potential determinant by sex were assessed by computing *P*-values from likelihood ratio tests and comparing models with and without the interaction terms.

A path analysis was conducted to evaluate the model fit of the hypothetical model and coefficients of each path. As a GLM analysis, the coefficients of each potential determinant at baseline on the overall LTPA at follow-up were estimated to compare the direction of effect with the hypothetical model (Figure 1). To evaluate the associations of potential determinants with the initiation phase of physical activity, path analyses were performed using changes of the categories of LTPA as an outcome in those who did not participate in physical activity at baseline. The associations of potential determinants with the maintenance phase of physical activity were evaluated in those who participated in physical activity 150 minutes or more per week at baseline. The coefficients from the initiation phase indicated how each determinant influenced the beginning of LTPA, and the coefficients from the maintenance phase indicated how each determinant affected the maintenance of LTPA. Sex stratification analysis was performed, and differences between the sexes were evaluated by comparing the chi-square of the unconstrained and structural weights models. We found that all of the models were significantly different according to sex (Δχ^2^/Δ degrees of freedom greater than 3.84).^28^ In all of the analyses, coefficients of standardized direct, indirect, and total effects were estimated to compare the parameters by removing scaling effects. The following indices of fit were considered a good fit: the goodness of fit index (GFI) ≥0.90, the comparative fit index (CFI) ≥0.90, and the root mean square error of approximation (RMSEA) <0.05. The generalized least squares method was used in the analysis.

In the obesity-related factors, WHR was used as a main indicator of obesity, because WHRs have clearer linear associations with LTPA than a BMI (Table 1). In social and cultural factors, although both social support and social capital act as important components of building social relationships, social support was used as a main indicator because social capital has not been sufficiently reported on to define its association with physical activity.^29^

**Table 1.** Least-square means (lsmeans) and beta estimates of the overall duration of leisure-time physical activity (min/week) at the follow-up by potential determinants at baseline according to level of LTPA at baseline, Korea, HEXA-G 2005–2016

	Total population, (*N* = 54,359)			Non-participation at baseline (*N* = 24,618)			≥150 min/week at baseline (*N* = 24,618)		

	%	Lsmean^a^	β (se)^a^	%	Lsmean^a^	β (se)^a^			
Sex									
Men	33.7	219.6	(ref)	30.4	123.6	(ref)	37.3	311.8	(ref)
Women	66.3	178.8	−40.6 (2.2)^***^	69.6	106.5	−17.1 (2.6)^***^	62.7	264.4	−47.4 (3.8)^***^
Age									
40–49	32.7	175.2	(ref)	35.5	106.4	(ref)	28.3	261.1	(ref)
50–59	42.1	202.4	27.2 (2.4)^***^	40.4	115.4	8.9 (2.7)^***^	44.1	288.0	26.9 (4.4)^***^
60–69	25.2	220.0	44.9 (2.8)^***^	24.1	123.4	17.0 (3.2)^**^	27.6	315.2	54.0 (4.9)^***^
*P* for trend			<0.001			<0.001			<0.001
*P* for interaction with sex			<0.001			<0.001			<0.001
Education									
≤Middle school graduate	30.2	176.0	(ref)	34.6	104.4	(ref)	27.3	277.7	(ref)
High school graduate	43.3	206.5	30.4 (2.6)^***^	42.1	120.7	16.4 (2.9)^***^	44.6	291.6	14.0 (4.6)^**^
≥College graduate	26.5	211.0	35.0 (3.0)^***^	23.3	120.3	15.9 (3.5)^***^	28.2	291.1	13.4 (5.0)^*^
*P* for trend			<0.001			<0.001			0.126
*P* for interaction with sex			<0.001			<0.001			0.007
Income per month, won									
<2.0 million	30.3	178.7	(ref)	34.3	108.1	(ref)	27.2	277.5	(ref)
2.0–4.0 million	44.1	204.2	25.6 (2.5)^**^	43.9	116.6	8.5 (2.8)^**^	44.7	292.6	15.1 (4.6)^**^
≥4.0 million	25.6	215.4	36.7 (2.9)^***^	21.8	123.6	15.5 (3.5)^***^	28.1	291.0	13.5 (5.2)^**^
*P* for trend			<0.001			<0.001			0.012
*P* for interaction with sex			0.193			0.387			0.479
Chronic diseases									
The number of chronic diseases			8.0 (1.5)^***^			5.7 (1.8)^**^			3.8 (2.5)
*P* for interaction with sex			<0.001			0.006			<0.001
Hypertension (yes versus no)	19.4	208.9	12.4 (2.7)^***^	18.0	121.5	8.1 (3.2)^*^	21.4	296.4	10.7 (4.6)^**^
*P* for interaction with sex			<0.001			<0.001			<0.001
Diabetes (yes versus no)	6.7	218.5	20.9 (4.2)^***^	5.8	132.6	18.9 (5.2)^***^	7.8	295.3	7.9 (6.9)
*P* for interaction with sex			0.018			0.147			0.133
CVDs (yes versus no)	3.8	198.2	−1.0 (5.5)	3.7	120.6	5.8 (6.4)	4.2	289.3	1.3 (9.2)
*P* for interaction with sex			0.031			0.635			0.162
Cancer (yes versus no)	3.5	215.3	16.6 (5.6)^**^	3.2	118.7	3.7 (6.8)	4.2	302.2	14.6 (9.1)
*P* for interaction with sex			0.141			0.249			0.527
Stress									
Not at all	57.2	211.1	(ref)	53.4	119.1	(ref)	61.7	297.9	(ref)
Often	35.2	185.4	−25.7 (2.2)^***^	37.1	110.4	−8.8 (2.6)^***^	32.4	274.6	−23.3 (4.0)^***^
Frequent	7.6	165.0	−46.1 (4.0)^***^	9.5	108.4	−10.8 (4.2)^*^	5.9	253.1	−44.8 (7.9)^***^
*P* for trend			<0.001			<0.001			<0.001
*P* for interaction with sex			0.406			0.640			0.245
Self-rated health									
Poor	16.4	164.8	(ref)	19.6	103.4	(ref)	13.1	259.6	(ref)
Normal	43.8	189.0	24.3 (3.0)^***^	46.0	114.9	11.5 (3.2)^***^	41.1	275.1	15.5 (5.8)^**^
Good	39.8	222.6	57.8 (3.1)^***^	34.4	121.5	18.1 (3.4)^***^	45.7	306.4	46.8 (5.7)^***^
*P* for trend			<0.001			<0.001			<0.001
*P* for interaction with sex			0.025			0.025			0.406
Score of depression symptom			−11.2 (0.7)^***^			−4.5 (0.8)^***^			−9.7 (4.7)^***^
*P* for interaction with sex			0.511			0.807			0.527
Social supports									
The number of social supports, mean (SD)			18.9 (2.6)^***^			7.9 (2.8)^**^			12.7 (4.9)^*^
*P* for interaction with sex			0.941			0.559			0.230
Having someone to confide (yes versus no)	91.7	201.8	29.1 (3.8)^***^	90.5	116.5	13.9 (4.1)^***^	92.7	289.6	19.5 (7.0)^**^
*P* for interaction with sex			0.803			0.502			0.342
Taking emotional caring (yes versus no)	96.1	200.1	22.1 (5.4)^***^	95.5	115.4	6.1 (5.8)	96.9	288.6	13.9 (10.4)
*P* for interaction with sex			0.791			0.974			0.257
Social capital (z-score)									
Total of social capital			5.4 (0.5)^***^			2.0 (0.6)^***^			4.5 (0.8)^***^
*P* for interaction with sex			0.205			0.594			0.269
Number of family member thought to be ​ very close			4.1 (1.2)^***^			0.9 (1.2)			4.6 (1.8)^**^
*P* for interaction with sex			0.810			0.531			0.698
Frequency of contact with family member ​ thought to be very close			5.2 (1.0)^***^			2.1 (1.2)			4.9 (1.9)^**^
*P* for interaction with sex			<0.001			0.101			0.004
Number of relatives, friends, colleagues, ​ and neighbors thought to be very close			6.2 (1.0)^***^			3.8 (1.4)^**^			4.4 (1.7)^**^
*P* for interaction with sex			0.094			0.229			0.238
Frequency of contact with relatives, friends, ​ colleagues, and neighbors thought to be very close			11.5 (1.3)^***^			3.4 (1.3)^**^			8.2 (1.9)^***^
*P* for interaction with sex			0.224			0.386			0.202
Smoking status, %									
Non-smoker ​ (never + quit smoking more than 10 years)	82.5	206.6	(ref)	81.5	118.7	(ref)	83.0	291.6	(ref)
Current smoker ​ (current + quit smoking less than 10 years)	17.5	176.6	−30.0 (3.3)^***^	18.5	105.9	−12.8 (3.9)^**^	17.0	275.8	−15.9 (5.7)^**^
*P* for interaction with sex			0.317			0.664			0.966
Waist-hip ratio^b^									
1Q	26.9	207.9	4.3 (3.0)	25.7	110.4	−7.9 (3.5)^*^	27.4	299.8	10.5 (5.1)^*^
2Q	23.1	203.6	(ref)	22.3	118.3	(ref)	23.7	289.3	(ref)
3Q	23.5	199.7	−3.9 (3.0)	23.5	116.6	−1.7 (3.5)	23.6	287.6	−1.7 (5.3)
4Q	26.5	186.2	−17.5 (3.0)^***^	28.5	115.4	−2.9 (3.4)	25.3	274.1	−15.2 (5.3)^**^
*P* for trend			<0.001			0.215			<0.001
*P* for interaction with sex			0.001			0.035			0.018
BMI, WHO classification, kg/m^2^									
<18.5	1.7	161.9	−40.1 (8.0)^***^	2.1	86.3	−29.0 (8.3)^***^	1.2	282.8	−7.5 (16.5)
18.5–24.9	66.4	202.0	(ref)	65.4	115.3	(ref)	66.8	290.3	(ref)
25.0–29.9	29.2	197.2	−4.8 (2.3)^*^	29.2	117.0	1.7 (2.7)	29.7	284.6	−5.7 (4.1)
≥30	2.7	176.8	−25.2 (6.4)^***^	3.3	109.0	−6.3 (6.8)	2.2	275.3	−15.0 (12.4)
*P* for trend			0.043			0.246			0.114
*P* for interaction with sex			<0.001			0.003			<0.001

BMI, body mass index; HEXA-G, health examinees gem study; CVDs, cardiovascular diseases; LTPA, leisure-time physical activity; WHO, World Health Organization.

The proportion of missing values was not presented when they were less than 5%.

^*^P < 0.05; ^**^P < 0.01; ^***^P < 0.001.

^a^Adjusted for sex and age.

^b^A cutoff values in men: 0.86, 0.89, and 0.92; women: 0.79, 0.84, and 0.88.

A GLM was conducted with SAS version 9.4 (SAS Inc., Cary, NC, USA), and path analysis was performed with AMOS 24.0.0 (SPSS Inc., Chicago, IL, USA). Statistical significance was verified at a *P* < 0.05 level.

## RESULTS

Among the 54,359 eligible participants (18,382 men and 35,971 women), the mean age at baseline was 54.7 (standard deviation [SD], 8.2) years for men and 52.8 (SD, 7.5) years for women. In Table 1, the associations between potential determinants at baseline and the overall duration of LTPA at follow-up are presented according to the level of physical activity at baseline. Women had a lower level of LTPA than men. Between both levels of physical activity, most potential determinants on LTPA were consistently associated with existing evidence as presented in eTable 1. These associations were consistently found in the analysis, including participants with missing values (eTable 3).

The results of the path analysis between the determinants and overall LTPA are presented in eFigure 2 and eTable 4. Most of the associations between the potential determinants had the same direction when compared to the hypothetical model (Figure 1), except for the associations between WHR and self-rated health (nonsignificant) and between WHR and smoking (inverse).

**Figure 2.**
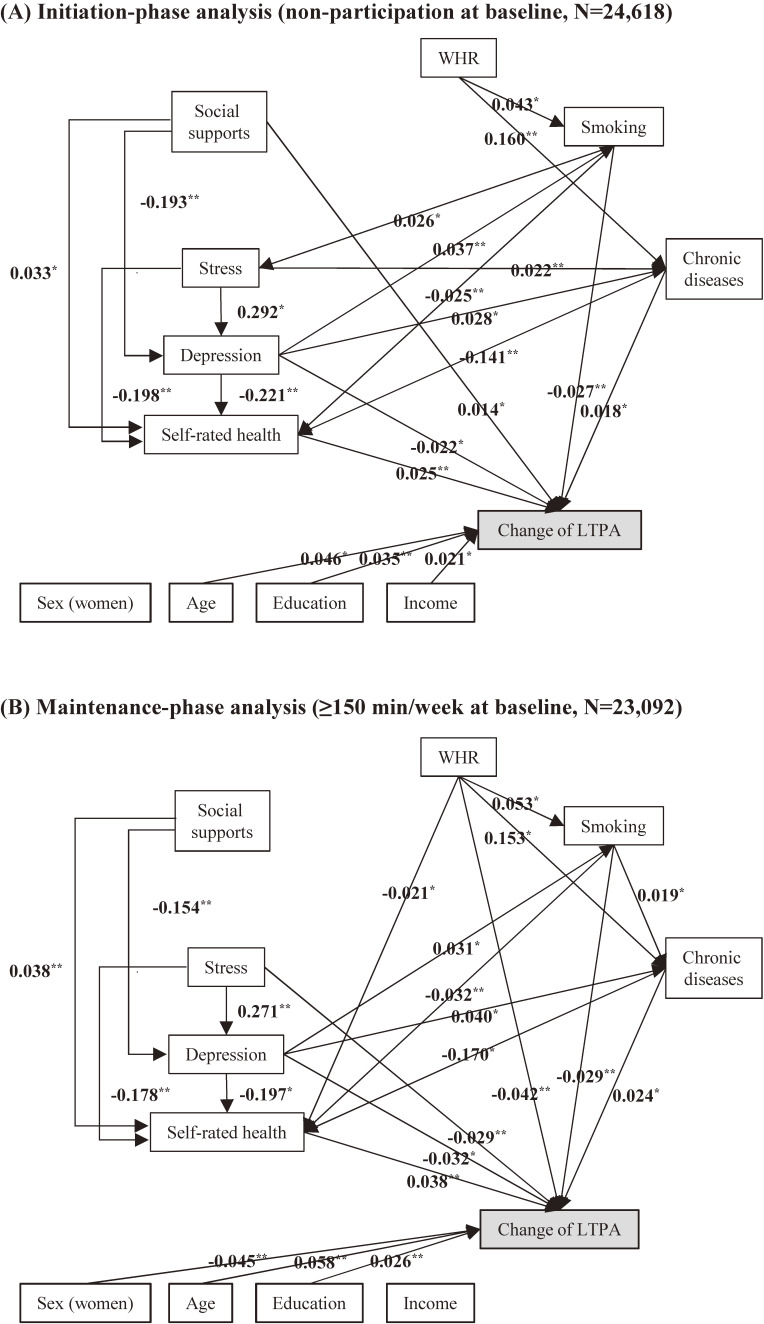
Standardized coefficients of the direct effect of potential determinants at baseline and the duration of leisure-time physical activity in the initiation and maintenance phases. Only significant associations are presented. Model fit indices: GFI: 0.998, CFI: 0.980, and RMSEA: 0.036 in initiation phase; GFI: 0.999, CFI: 0.991, and RMSEA: 0.042 in maintenance phase. ^*^P < 0.05; ^**^P < 0.01; ^***^P < 0.001. LTPA, leisure-time physical activity; WHR, waist-to-hip ratio.

Figure 2 shows the direct effect of the potential determinants on (A) initiation and (B) maintenance of the LTPA. The coefficient of direct, indirect, and total effect according to sex are presented in eTable 5. All indices of fit were satisfied as described in the footnote of Figure 2. In the total population, age, education, chronic diseases, smoking, depression symptoms, and self-rated health were significantly associated with both initiation and maintenance phases. Income (*B* = 0.025) and social supports (*B* = 0.019) were associated only with the initiation phase. Waist-to-hip ratio (*B* = −0.042) and stress (*B* = −0.029) were associated only with the maintenance phase. After stratifying by sex, the significant effects of education (*B* = 0.101), chronic diseases (*B* = 0.036), and smoking (*B* = −0.039) were found only in men. The initiation phase-specific effects of income and social supports and the maintenance phase-specific effects of stress were found only in women.

In most of the determinants, it was estimated that approximately 15% of the total effects were attributed to indirect effects. The significant indirect effects were found in education, income, chronic diseases, social supports, smoking, stress, and depression symptoms in both phases in the total population. With regard to the causal path between the determinants, the association between stress and smoking was only found in the initiation phase (*B* = 0.026), whereas the associations between stress and WHR (*B* = 0.014) and between smoking and chronic diseases (*B* = 0.019) were only found in the maintenance phase. Those patterns of associations with the initiation and maintenance of LTPA were consistently found in analysis, using BMI as a substitute for WHR (eTable 6). In the analysis using social capital as a substitute of social support, there were significant associations in both the initiation and maintenance phases.

## DISCUSSION

This longitudinal study established a hypothetical model between potential determinants and LTPA, based on previous studies, and comprehensively evaluated the phase-specific associations of each determinant. Age, education, chronic diseases, smoking, depression symptoms, and self-rated health had significant associations with the duration of LTPA in both the initiation and maintenance phases. Initiation-specific determinants, such as income and social supports, and maintenance-specific determinants, such as WHR, were found. Most of the contributions to the indirect effects were smaller than those of the direct effects, but they contributed in some degree to the total effect.

To the best of our knowledge, this is the first study to construct a hypothetical model of determinants of physical activity in a large-scale cohort, based on previously established studies. This approach has the advantage of evaluating several determinants at once in a model through different pathways at each phase. It was well-known that income is a positive determinant of physical activity. The present study confirmed that the direct effects of income were only significant in the initiation and maintenance phases. After stratifying by sex, we further found that the indirect effect was significant in both men and women, but the direct effect was significant only in women. These findings confirm existing evidence that the attainment of higher socioeconomic status indirectly improves health through economic conditions, social and psychological resources, and a healthy lifestyle in both sexes,^30^ and newly suggests that the direct effects only affect women. In social support, only a few studies suggested the initiation-specific total effects of social supports on vigorous physical activity in women.^31^ The present study further confirmed that those initiation-specific effects were mostly derived from direct effects in women. One of the main reasons for these results would be that women are more sensitive to social cues in adopting appropriate behavior than are men.^32^ In obesity-related factors, it had been reported that obese participants were less likely to initiate^20^^,^^33^^,^^34^ and maintain^31^^,^^33^^–^^36^ physical activity. The present study showed that there were maintenance-specific effects of obesity and these effects were derived from direct effects. According to Boutelle et al,^34^ obesity had a short-term effect on the initiation phase in less than 2 years. It is possible that only maintenance-specific effects of obesity could be found in the present study because the median follow-up duration was 4.2 years. These findings suggest that it is important to evaluate the direct and indirect effects of each determinant separately and that different determinant sets should be considered in phase- or gender-specific settings.

The opposite association of age between the results in the present and previous studies could be thought to be a result of the birth cohort effect. In the present study, physical activity levels were lower in the relatively younger group born more recently (>1960s) than in the group born earlier (<1950s) (eTable 7), which is in line with previous evidence, suggesting that physical activity has decreased in the younger generations.^37^ At the same time, it was also observed that physical activity levels decreased with age in each birth cohort born in the 1950s or earlier in this study. Thus, the positive effects of age on physical activity in the present study may have been caused by a greater birth cohort effect than an age effect. In Korea, these birth cohort effects could also have been reinforced by the rapid social and economic growth experienced since the 1960s.^38^

Chronic diseases, frequently reported diseases such as hypertension, diabetes, CVD, and cancer,^15^^,^^16^^,^^19^^,^^20^ for which exercise is recommended for treatment and rehabilitation^39^^–^^42^ were included in the present study. These diseases had positive direct effects, which were opposite from the results of previous studies. Given the nature of self-reported data, these differences may be derived from overestimation of the LTPA by the participants with chronic diseases, due to social desirability bias,^43^^,^^44^ which is known to have a stronger effect in East Asian countries than in Western countries,^45^ where most of the previous studies have been conducted. The phase-specific effects of chronic diseases should be confirmed through more accurate data on physical activity, such as accelerometer measurements.

In most of the determinants, indirect effects were estimated to contribute to about 10–20% of the total effect. Self-rated health or chronic diseases could be the most contributing factor in the indirect effect because most of the other determinants pass through them in the model. Further studies of the impact of these factors on physical activity may lead to more effective clinical benefits for disease rehabilitation or better prognosis.

The present study has some limitations. First, the LTPA variables were collected using a self-reported questionnaire. However, the validity and reliability of the physical activity questionnaire were confirmed by comparing the results with objectively measured physical activity.^26^ Second, the follow-up rate was as low as 46.2%, and those who participated in the follow-up showed a statistical difference in the distribution of determinants and level of physical activity (eTable 8), although these differences were not large. Thus, the results of the present study need to be generalized with care. Third, we could not consider the days of physical activity lasting more than 10 minutes. However, recent evidence suggests that the association of moderate to vigorous physical activity (MVPA) with reduction in mortality was independent of bout of physical activity.^46^ The results of the present study were also unchanged after excluding participants who performed fewer than 10 minutes of physical activity on average (eTable 9). Fourth, some other determinants could not be assessed, such as psychological and environmental factors. For example, self-efficacy is known as the one of the important determinants of physical activity^47^ and acts as a moderator among social support, stress, and perceived health with LTPA.^48^^–^^51^ Based on previous results, the magnitude of the indirect effects of those potential determinants was expected to increase slightly, from approximately 0.01 to 0.04, when self-efficacy was included in our hypothetical model. Further studies of longitudinal design with important endogenous factors will be needed to evaluate their comprehensive effects on physical activity.

By constructing a hypothetical model between potential determinants and LTPA based on the results of previous longitudinal studies, initiation-specific determinants, such as social support, and maintenance-specific determinants, such as WHR according to sex, were found. In addition, each potential determinant had a relatively different direct or indirect effect on the LTPA. Our results will help in designing appropriate interventions to promote LTPA by providing the structure and specific effects of important determinants on initiation and maintenance of physical activity.

## APPENDIX A. SUPPLEMENTARY DATA

eTable 1. Evidence for direct and indirect associations between potential determinants and level of physical activity

eTable 2. Change in leisure-time physical activity between baseline and follow-up

eTable 3. Least square means (lsmeans) and beta estimates of the duration of overall leisure-time physical activity (min/week) at follow-up by potential determinants at baseline according to level of LTPA at baseline including those who have missing data in determinants and physical activity, Korea, HEXA-G 2005–2016

eTable 4. Summary of standardized direct, indirect and total effects of potential determinants on the duration leisure-time physical activity at follow-up

eTable 5. Summary of standardized direct, indirect, and total effects of potential determinants on the initiation and maintenance of leisure-time physical activity

eTable 6. Summary of standardized direct, indirect, and total effects of body on the initiation and maintenance of leisure-time physical activity

eTable 7. Distribution of participation in physical activity at follow-up according to age at baseline and year of birth

eTable 8. Distribution of potential determinants and leisure-time physical activity according to the inclusion and lost-to-follow-up status

eTable 9. Summary of standardized direct, indirect and total effects according to different definitions of non-participating group

eFigure 1. Inclusion and exclusion criteria of study population

eFigure 2. Standardized coefficients of direct effect of potential determinants at baseline and the duration of overall leisure-time physical activity at follow-up
