# Supplementary material for: Relative Effects of Demographic, Psychological, Behavioral, and Social Factors on the Initiation and Maintenance of Leisure-time Physical Activity: Results From a Confirmatory Path Analysis in a Longitudinal Study
Source: J Epidemiol. 2021 Nov 5;31(11):557–65. doi: 10.2188/jea.JE20200073 (PMC8502832; doi:10.2188/jea.JE20200073)
Supplement: Supplementary file 1 [file je-31-557-s001.pdf]

eTable 1. Evidence for direct and indirect associations between potential determinants and level of physical activity

Independent variable	Dependent variable	Direction of association (references)			Number of studies (- / Null / +)
		Negative	Null	Positive	
Direct effect					
Sex (women)	Physical activity	[1-7]	[8-10]	[11, 12]	7 / 3 / 2
Age	Physical activity	[5-7, 13-18]	[3, 9, 12, 19-22]	[1]	9 / 7 / 1
Education	Physical activity	[7, 21]	[3, 9, 18, 19, 22-25]	[1, 4-6, 12-14, 17, 20, 26-30]	2 / 8 / 14
Income	Physical activity		[3, 5, 9, 18, 19]	[1, 15, 16, 28, 29, 31, 32]	0 / 5 /7
Smoking	Physical activity	[1, 3, 10, 12, 13, 21, 26, 28]	[4, 5, 14, 17, 25, 27, 33]		8 / 7 / 0
Obesity	Physical activity	[1, 3, 5-7, 13, 14, 17, 21, 25, 28, 30]	[4, 10]		12 / 2 /0
Social supports	Physical activity		[1, 5, 19, 34]	[6, 13, 15, 26, 35-38]	0 / 4 / 9
Self-rated health	Physical activity	[5, 35]	[17]	[6, 12, 21, 25, 28, 32]	2 / 1 / 6
Depression	Physical activity	[3, 20, 30, 39, 40]	[32]		5 / 1 / 0
Chronic diseases	Physical activity	[7, 14, 16, 20, 28, 41, 42]	[3, 24]	[43, 44]	7 / 2 / 2
Stress	Physical activity	[45][a]			
Indirect effect[b]					
Stress	Depression			[46][c]	
Stress	Obesity			[47][c]	
Stress	Chronic diseases			[48, 49][c]	
Stress	Self-rated health	[50-56]	[57]		7 / 1 / 0
Depression	Smoke			[58][c]	
Depression	Chronic diseases			[59, 60][c]	
Depression	Self-rated health	[61-63]	[57]		3 / 1 / 0
Obesity	Chronic diseases			[64-66][c]	
Obesity	Self-rated health	[54, 56, 61, 63, 67]	[62, 68]		5 / 2 / 0
Obesity	Smoking	[69-71]	[72]		3 / 1 / 0
Smoking	Chronic diseases			[73-77][c]	
Smoking	Stress			[78][c]	
Smoking	Self-rated health	[54, 56, 61-63, 68, 79]	[57, 80]		7 / 2 / 0
Social supports	Depression	[81-83]	[84]		3 / 1 / 0
Social supports	Smoking	[85-89]	[90-93]	[94]	5 / 4 / 1
Social supports	Self-rated health		[51, 57]	[50, 52, 53, 56]	0 / 2 / 4
Chronic diseases	Self-rated health	[54, 56, 62, 63]	[57]		4 / 1 / 0

[a]Results from systematic reviews of prospective studies
[b]Effects of demographic factors, such as sex, age, education and income, on other potential determinants are not summarized because they were used as confounders in the hypothetical model
[c]Results from a meta-analysis of prospective studies

eTable 2. Change in leisure-time physical activity between baseline and follow-up

Analysis	Level of physical activity		Change	N	%
	At baseline	At follow-up			
Initiation-phase (N=24,618)	None	None	Stable (0 level)	14,568	59.2
		<150 min/week	Increasing (+1 level)	2,867	11.6
		≥150 min/week	Increasing (+2level)	7,183	29.2
Maintenance-phase (N=23,092)	≥150 min/week	None	Decreasing (-2 level)	5,775	25.0
		<150 min/week	Decreasing (-1 level)	2,241	9.7
		≥150 min/week	Stable (0 level)	15,076	65.3

eTable 3. Least square means (lsmeans) and beta estimates of the duration of overall leisure-time physical activity (min/week) at follow-up by potential determinants at baseline according to level of LTPA at baseline including those who have missing data in determinants and physical activity, Korea, HEXA-G 2005–2016

	Total population (N=62,788)			Non-participation at baseline (N=27,626)			≥150 min/week at baseline (N=25,487)		
	%	Lsmean[a]	β (se)[a]	%	Lsmean[a]	β (se)[a]	%	Lsmean[a]	β (se)[a]
Sex									
Men	33.8	219.8	(ref)	30.7	124.8	(ref)	37.2	311.3	(ref)
Women	66.2	177.8	-41.7 (2.2)***	69.3	105.9	-18.9 (2.4)***	62.8	263.5	-47.8 (3.6)***
Age, years									
40–49	32.0	175.4	(ref)	34.8	107.1	(ref)	27.8	231.1	(ref)
50–59	42.4	202.5	27.0 (2.3)***	40.8	116.4	9.3 (2.6)***	44.2	312.4	27.6 (4.3)***
60–69	25.6	218.6	43.2 (2.6)***	24.4	122.5	15.4 (3.0)***	28.0	288.7	51.3 (4.7)***
p for trend			<0.001			<0.001			<0.001
p for interaction with sex			<0.001			<0.001			<0.001
Education									
≤Middle school graduate	30.6	176.3	(ref)	35.0	105.4	(ref)	27.8	276.5	(ref)
High school graduate	42.5	206.2	23.5 (2.4)***	41.4	120.4	15.1 (2.7)***	44.1	291.5	14.9 (4.3)***
≥College graduate	25.6	211.0	33.6 (2.8)***	22.7	120.6	15.2 (3.3)***	27.4	290.7	14.1 (5.0)
p for trend			<0.001			<0.001			0.005
p for interaction with sex			<0.001			<0.001			0.005
Income per month, won									
<2.0 million	27.9	179.1	(ref)	31.5	109.0	(ref)	25.4	277.6	(ref)
2.0–4.0 million	40.6	204.7	25.9 (2.5)***	40.4	117.7	8.6 (2.8)**	41.7	292.0	14.4 (4.5)**
≥4.0 million	23.4	215.6	36.8 (2.9)***	20.1	123.8	14.7 (3.4)***	26.1	290.8	13.2 (5.1)**
Unknown	8.1	189.0	10.2 (3.9)**	8.0	108.6	-0.4 (4.5)	6.9	280.6	3.0 (7.4)
p for trend			<0.001			<0.001			0.007
p for interaction with sex			0.308			0.756			0.448
Chronic diseases									
The number of chronic diseases			8.4 (1.4)***			4.9 (1.7)**			5.0 (2.4)*
p for interaction with sex			<0.001			0.004			<0.001
Hypertension (yes *versus* no)	19.5	208.9	12.6 (2.6)***	18.1	120.7	6.8 (3.0)*	21.4	296.7	12.1 (4.4)**
p for interaction with sex			<0.001			0.002			<0.001
Diabetes (yes *versus* no)	6.7	217.7	20.2 (4.0)***	5.9	130.7	16.6 (4.9)***	7.8	296.3	9.7 (6.5)
p for interaction with sex			0.002			0.095			0.042
CVD (yes *versus* no)	3.8	199.2	0.4 (5.2)	3.7	121.6	6.5 (6.0)	4.1	289.0	1.7 (8.8)
p for interaction with sex			0.005			0.936			0.083
Cancer (yes *versus* no)	3.5	214.4	16.2 (5.3)**	3.2	116.5	1.2 (6.4)	4.1	301.2	14.4 (8.7)
p for interaction with sex			0.273			0.396			0.301
Stress									
Not at all	56.0	210.7	(ref)	52.4	119.2	(ref)	60.9	297.0	(ref)
Often	35.1	185.6	-25.0 (2.1)***	37.2	111.0	-8.2 (2.4)***	32.5	274.5	-22.5 (3.8)***
Frequent	7.8	164.8	-45.6 (3.7)***	9.7	107.5	-11.7 (3.9)**	6.0	252.7	-44.3 (7.4)***

p for trend			<0.001			<0.001			<0.001
p for interaction with sex			0.730			0.961			0.362
Self-rated health									
Poor	16.5	163.3	(ref)	19.9	103.3	(ref)	13.2	145.6	(ref)
Normal	43.6	189.3	26.0 (2.8)***	45.9	115.2	12.0 (3.0)***	41.2	170.6	18.7 (5.4)***
Good	39.0	222.5	59.0 (2.9)***	33.7	121.9	18.7 (3.2)***	45.2	196.4	48.8 (5.4)***
p for trend			<0.001			<0.001			<0.001
p for interaction with sex			<0.001			0.012			0.623
Score of depression symptom						-4.3 (0.8)***			-9.0 (1.3)***
p for interaction with sex						0.875			0.821
Social supports									
The number of social supports, mean (SD)			18.5 (2.4)***			8.0 (2.6)**			11.9 (4.5)**
p for interaction with sex			0.867			0.889			0.454
Having someone to confide (yes *versus* no)	90.5	201.6	28.3 (3.4)***	89.6	116.9	13.5 (3.8)**	92.1	288.9	17.4 (6.5)**
p for interaction with sex			0.678			0.841			0.669
Taking emotional caring (yes *versus* no)	95.1	199.9	22.5 (4.9)***	94.9	115.7	7.3 (5.3)	96.3	288.0	15.0 (9.5)
p for interaction with sex			0.893			0.995			0.299
Social capital (z-score)									
Total of social capital			5.2 (0.4)***			1.8 (0.5)***			4.5 (4.2)***
p for interaction with sex			0.070			0.525			0.138
No. of family member thought to be very close			4.1 (1.0)***			0.4 (0.4)			5.4 (1.7)**
p for interaction with sex			0.613			0.262			0.760
Frequency of contact with family member thought to be very close			4.6 (1.0)***			1.8 (1.1)			3.9 (1.8)*
p for interaction with sex			<0.001			0.040			0.003
No. of relatives, friends, colleagues, and neighbors thought to be very close			6.6 (1.0)***			3.3 (1.3)*			5.3 (1.6)***
p for interaction with sex			0.233			0.285			0.291
Frequency of contact with relatives, friends, colleagues, and neighbors thought to be very close			10.8 (1.0)***			4.0 (1.2)***			7.3 (1.8)***
p for interaction with sex			0.144			0.175			0.114
Smoking status, %									
Non-smoker (never + quit smoking more than 10 years)	81.0	205.4	(ref)	80.6	118.6	(ref)	82.3	290.2	(ref)
Current smoker (current + quit smoking less than 10 years)	17.0	175.6	-31.1 (3.1)***	18.1	104.2	-14.4 (3.7)***	16.7	276.9	-13.3 (5.5)*
p for interaction with sex			0.538			0.721			0.717
Waist-hip ratio[b]									
1Q	21.3	207.8	4.6 (2.8)	25.4	111.1	-6.9 (3.3)*	27.1	300.0	11.2 (4.9)*
2Q	25.3	203.0	(ref)	22.2	118.0	(ref)	23.7	288.8	(ref)
3Q	26.4	199.9	-2.9 (2.8)	23.5	117.1	-1.0 (3.3)	23.7	286.8	-2.0 (5.0)
4Q	26.5	185.2	-17.8 (2.8)***	28.6	115.4	-2.6 (3.2)	25.3	272.0	-16.8 (5.0)***
p for trend			<0.001			0.260			<0.001

p for interaction with sex			<0.001			0.052			0.009
BMI, WHO classification (kg/m^2)									
<18.5	1.7	161.6	-40.6 (7.6)***	2.1	87.8	-28.1 (7.9)***	1.2	285.9	-3.6 (15.9)
18.5–24.9	66.1	201.8	(ref)	65.2	115.8	(ref)	66.7	289.5	(ref)
25.0–29.9	29.4	196.6	-5.2 (2.2)*	29.4	116.8	1.0 (2.5)	29.8	284.3	-5.2 (3.8)
≥30	2.7	173.7	-28.0 (6.1)***	3.3	106.7	-9.1 (6.4)	2.2	269.9	-19.6 (11.7)
p for trend			0.011			0.467			0.069
p for interaction with sex			<0.001			0.013			<0.001

CVD, cardiovascular diseases; LTPA, leisure-time physical activity.

The proportion of missing values was not presented when they were less than 5%

*p<0.050; **p<0.010; ***p<0.001

[a]Adjusted for sex and age

[b]A cutoff values were 0.86, 0.89 and 0.92 in men and 0.79, 0.84 and 0.88 in women

eTable 4. Summary of standardized direct, indirect and total effects of potential determinants on the duration leisure-time physical activity at follow-up

	Overall LTPA		
	Direct effect	Indirect effect	Total effect
Total[a] (N=54,359)			
Sex (women)	-0.030**	0.020**	-0.010*
Age	0.044*	0.006**	0.050*
Education	0.030*	0.011*	0.041*
Income	0.022*	0.007**	0.029*
Chronic diseases	0.017**	-0.005*	0.012**
Social supports	0.014**	0.007*	0.022**
WHR	-0.020**	0.000	-0.020*
Smoking	-0.031*	-0.001*	-0.032*
Stress	-0.018*	-0.016*	-0.034*
Depression symptom	-0.030*	-0.007*	-0.037**
Self-rated health	0.032*		0.032*
Men (N=18,383)			
Age	0.056*	0.017**	0.074**
Education	0.075**	0.011**	0.087*
Income	0.022*	0.007*	0.029**
Chronic diseases	0.030**	-0.010*	0.020*
Social supports	0.015	0.008*	0.022*
WHR	-0.012	0.000	-0.012
Smoking	-0.034**	-0.001*	-0.035**
Stress	-0.010	-0.018*	-0.028*
Depression symptom	-0.024*	-0.012**	-0.036*
Self-rated health	0.052**		0.052**
Women (N=35,976)			
Age	0.033*	-0.002	0.031**
Education	0.000	0.011**	0.011*
Income	0.021*	0.007*	0.028*
Chronic diseases	0.009	-0.003**	0.006
Social supports	0.014*	0.008*	0.022*
WHR	-0.025**	0.000	-0.025**
Smoking	-0.015**	-0.001*	-0.016**
Stress	-0.023*	-0.015*	-0.038*
Depression symptom	-0.034**	-0.005**	-0.039*
Self-rated health	0.021**		0.021**

LTPA, leisure-time physical activity; WHR, waist-to-hip ratio.
Differences between men and women were significant in duration ($\Delta\chi^2$/Δdegree of freedom=35.6)
*p<0.050; **p<0.010; ***p<0.001
[a]Model fit indices: GFI: 0.996, CFI: 0.972, and RMSEA: 0.049

eTable 5. Summary of standardized direct, indirect, and total effects of potential determinants on the initiation and maintenance of leisure-time physical activity

	Change of LTPA					
	Non-participation at baseline *Initiation phase*[a]			≥150 min/week at baseline *Maintenance phase*[b]		
	Direct effect	Indirect effect	Total effect	Direct effect	Indirect effect	Total effect
Total						
Sex (women)	-0.012	0.018**	0.006	-0.045**	0.026**	-0.019**
Age	0.041*	0.011*	0.052*	0.058**	0.003	0.061*
Education	0.041**	0.006**	0.047**	0.026**	0.015**	0.041*
Income	0.025**	0.007**	0.032**	0.015	0.005**	0.020*
Chronic diseases	0.020*	-0.003**	0.016	0.024**	-0.006**	0.018*
Social supports	0.019**	0.007**	0.026**	0.011	0.008*	0.019*
WHR	0.000	0.001	0.001	-0.042**	0.000	-0.042**
Smoking	-0.035**	-0.001**	-0.035**	-0.029**	-0.001**	-0.030**
Stress	-0.014	-0.014*	-0.028**	-0.029**	-0.018*	-0.046**
Depression symptom	-0.027*	-0.006**	-0.033*	-0.032*	-0.008**	-0.040*
Self-rated health	0.022**		0.022**	0.038**		0.038**
Men						
Age	0.060**	0.020**	0.080*	0.067**	0.014**	0.082**
Education	0.101*	0.006**	0.107**	0.058**	0.014*	0.072**
Income	0.024	0.007**	0.031*	0.018	0.004**	0.022
Chronic diseases	0.036**	-0.008**	0.028*	0.037*	-0.009**	0.028*
Social supports	0.015	0.007*	0.022*	0.019	0.010**	0.029*
WHR	0.001	0.002	0.003	-0.030*	0.001	-0.029*
Smoking	-0.039*	-0.002**	-0.041*	-0.035**	-0.001	-0.036**
Stress	-0.010	-0.018**	-0.027*	-0.015	-0.021*	-0.036**
Depression symptom	-0.015	-0.013*	-0.027*	-0.040*	-0.010**	-0.050*
Self-rated health	0.056**		0.056*	0.047**		0.047**
Women						
Age	0.028**	0.005	0.033**	0.049**	-0.006	0.043**
Education	0.008	0.007*	0.015**	0.002	0.014*	0.016
Income	0.026**	0.007**	0.032*	0.012	0.006*	0.018
Chronic diseases	0.011	-0.001	0.010	0.015	-0.005**	0.010
Social supports	0.021**	0.008*	0.029**	0.004	0.007**	0.011
WHR	-0.004	0.001	-0.003	-0.046*	0.000	-0.045*
Smoking	-0.016	0.001**	-0.016*	-0.015	-0.002*	-0.016
Stress	-0.017	-0.012*	-0.029*	-0.035**	-0.016**	-0.051**
Depression symptom	-0.033*	-0.002	-0.036**	-0.028**	-0.007**	-0.035**
Self-rated health	0.007		0.007	0.032**		0.032**

LTPA: leisure-time physical activity; WHR, waist-to-hip ratio.

Differences between men and women were significant in the initiation ($\Delta\chi^2$/Δdegree of freedom: 15.2) and maintenance phases ($\Delta\chi^2$/Δdegree of freedom: 16.3)

$^{*}p<0.050$; $^{**}p<0.010$; $^{***}p<0.001$

[a]Model fit indices: GFI: 0.998, CFI: 0.980, and RMSEA: 0.036 in initiation phase

[b]Model fit indices: GFI: 0.999, CFI: 0.991, and RMSEA: 0.042 in maintenance phase

eTable 6. Summary of standardized direct, indirect, and total effects of body on the initiation and maintenance of leisure-time physical activity

	Change of LTPA					
	Non-participation at baseline *Initiation phase*			≥150 min/week at baseline *Maintenance phase*		
	Direct effect	Indirect effect	Total effect	Direct effect	Indirect effect	Total effect
Obesity-related factors						
WHR	N=24,618			N=23,092		
	GFI: 0.998, CFI: 0.980, and RMSEA: 0.036			GFI: 0.999, CFI: 0.991, and RMSEA: 0.042		
	$\Delta\chi^2/\Delta df$: 15.2			$\Delta\chi^2/\Delta df$: 16.3		
Total	0.000	0.001	0.001	-0.042**	0.000	-0.042**
Men	0.001	0.002	0.003	-0.030*	0.001	-0.029*
Women	-0.004	0.001	-0.003	-0.046*	0.000	-0.045*
BMI	N=24,656			N=23,088		
	GFI: 0.998, CFI: 0.988, and RMSEA: 0.050			GFI: 0.999, CFI: 0.991, and RMSEA: 0.042		
	$\Delta\chi^2/\Delta df$: 17.9			$\Delta\chi^2/\Delta df$: 18.7		
Total	0.001	0.003	0.004	-0.023**	0.003*	-0.020*
Men	0.017	0.007*	0.024*	0.011	0.005*	0.016
Women	-0.011	0.002	-0.009	-0.045**	0.002	-0.043**
Obesity-related factors						
Social supports	N=24,618			N=23,092		
	GFI: 0.998, CFI: 0.980, and RMSEA: 0.036			GFI: 0.999, CFI: 0.991, and RMSEA: 0.042		
	$\Delta\chi^2/\Delta df$: 15.2			$\Delta\chi^2/\Delta df$: 15.1		
Total	0.019**	0.007**	0.026**	0.011	0.008*	0.019*
Men	0.015	0.007*	0.022*	0.019	0.010**	0.029*
Women	0.021**	0.008*	0.029**	0.004	0.007**	0.011
Social capital	N=23,959			N=22,515		
	GFI: 0.999, CFI: 0.994, and RMSEA: 0.035			GFI: 0.999, CFI: 0.993, and RMSEA: 0.038		
	$\Delta\chi^2/\Delta df$: 14.6			$\Delta\chi^2/\Delta df$: 16.2		
Total	0.016*	0.006**	0.022*	0.047**	0.005*	0.052**
Men	0.009	0.007*	0.016	0.056**	0.007*	0.063**
Women	0.019*	0.007*	0.025*	0.040**	0.004**	0.045**

LTPA: leisure-time physical activity; WHR, waist-to-hip ratio

$\Delta\chi^2$/Δdegree of freedom greater than 3.84 indicated that the moderating effect of sex was statistically significant

$^{*}p<0.050$; $^{**}p<0.010$; $^{***}p<0.001$

eTable 7. Distribution of participation in physical activity at follow-up according to age at baseline and year of birth

Age at baseline	Year of birth						Total	
	<1950s		1950s		>1960s			
	N	%[a]	N	%[a]	N	%[a]	N	%[a]
40-45					8,687	52.6	8,687	52.9
45-50			1,491	63.0	7,584	57.7	9,075	58.9
50-55			10,537	59.1	1,908	53.0	12,445	58.7
55-60	1,165	62.6	9,281	58.1			10,446	59.1
60-65	6,961	61.4	1,593	57.8			8,554	60.8
65-70	5,152	57.7					5,152	59.0
Total	13,278	60.4	22,902	58.8	18,179	54.8	54,359	57.9

[a]Proportion of participants who participated in leisure-time physical activity at follow-up

eTable 8. Distribution of potential determinants and leisure-time physical activity according to the inclusion and lost-to-follow-up status

	Total (N=139,344)				
	Included in FU (N=64,485, 46.2%)		Loss during FU (N=74,859, 53.8%)		
	N	%	N	%	*p*[a]
Potential determinants					
Sex					
Men	21,253	33.8	23,337	33.7	0.70
Women	41,535	66.2	45,815	66.3	
Age at baseline, years, mean (SD)	53.6 (7.8)		52.2 (8.1)		<0.01
40–49	20,077	32.0	27,758	40.1	<0.01
50–59	26,626	42.4	26,181	37.9	
60–69	16,085	25.6	15,213	22.0	
Education					
≤Middle school graduate	19,241	30.6	21,890	31.7	<0.01
High school graduate	26,687	42.5	28,619	41.4	
≥College graduate	16,055	25.6	17,297	25.0	
Income per month, won					
<2.0 million	17,514	27.9	19,350	28.0	<0.01
2.0–4.0 million	25,502	40.6	26,743	38.7	
≥4.0 million	14,710	23.4	15,040	21.7	
Unknown	5,062	8.1	8,019	11.6	
Social supports					
The number of social supports, mean (SD)	1.87 (0.41)		1.86 (0.44)		<0.01
Having someone to confide (yes)	56,812	90.5	61,758	89.3	<0.01
Taking emotional caring (yes)	59,689	95.1	65,035	94.0	<0.01
Social capital (z-score)					
Total of social capital, mean (SD)	0.05 (2.23)		-0.03 (2.28)		<0.01
Number of family member thought to be very close, mean (SD)	0.00 (0.99)		0.00 (1.01)		0.58
Frequency of contact with family member thought to be very close, mean (SD)	0.03 (0.99)		-0.03 (1.01)		<0.01
Number of relatives, friends, colleagues, and neighbors thought to be very close, mean (SD)	0.00 (1.03)		0.00 (0.97)		0.65
Frequency of contact with relatives, friends, colleagues, and neighbors thought to be very close, mean (SD)	0.01 (0.98)		-0.01 (1.02)		<0.01
Chronic diseases					
The number of chronic diseases, mean (SD)	0.33 (0.60)		0.30 (0.60)		<0.01
Hypertension (yes)	12,218	19.5	12,451	18.0	<0.01
Diabetes (yes)	4,192	6.7	4,285	6.2	<0.01
CVD (yes)	2,356	3.8	2,136	3.1	<0.01
Cancer (yes)	2,209	3.5	2,039	2.9	<0.01
Stress					
Not at all	35,132	56.0	37,437	55.0	<0.01
Often	22,063	35.1	24,852	36.5	
Frequent	4,869	7.8	5,726	8.4	
Self-rated health					
Poor	10,363	16.5	11,134	16.3	<0.01
Normal	27,406	43.6	30,282	44.4	
Good	24,461	39.0	26,823	39.3	
Score of depressive symptom, mean (SD)	1.69 (1.41)		1.76 (1.47)		<0.01
BMI (kg/m2), mean (SD)	23.9 (2.86)		23.9 (2.94)		0.79
Waist-hip ratio, mean (SD)	0.86 (0.07)		0.86 (0.07)		0.55
Smoking status, %					
Non-current (never + quit smoking more than 10 years)	50,888	81.0	53,482	77.3	<0.01
Current (current + quit smoking less than 10 years)	10,674	17.0	13,938	20.2	
Physical activity at baseline					
Leisure-time physical activity					
Duration (min/week), mean (SD)	174.0 (243.8)		149.9 (229.8)		<0.01

FU, follow-up; CVD, cardiovascular diseases; LTPA, leisure-time physical activity.
The proportion of missing values was not presented when they were less than 5%
[a]*p* value was estimated by the chi-square test for categorical variables and the t-test for continuous variables

eTable 9. Summary of standardized direct, indirect and total effects according to different definitions of non-participating group

	Assessing LTPA without 10 minute/time cutoff			Assessing those who participating in LTPA <10 minutes/time to non-participation group		
	Direct effect	Indirect effect	Total effect	Direct effect	Indirect effect	Total effect
Total[a] (N=54,359)						
Sex (women)	-0.030**	0.020**	-0.010*	-0.030**	0.020**	-0.010*
Age	0.044*	0.006**	0.050*	0.044*	0.006**	0.050*
Education	0.030*	0.011*	0.041*	0.030*	0.011*	0.041*
Income	0.022*	0.007**	0.029*	0.022*	0.007**	0.029*
Chronic diseases	0.017**	-0.005*	0.012**	0.017**	-0.005*	0.012**
Social supports	0.014**	0.007*	0.022**	0.014**	0.007*	0.022**
WHR	-0.020**	0.000	-0.020*	-0.020**	0.000	-0.020*
Smoking	-0.031*	-0.001*	-0.032*	-0.031*	-0.001*	-0.032*
Stress	-0.018*	-0.016*	-0.034*	-0.018*	-0.016*	-0.034*
Depression symptom	-0.030*	-0.007*	-0.037**	-0.030*	-0.007*	-0.037*
Self-rated health	0.032*		0.032*	0.031*		0.031*

LTPA, leisure-time physical activity; WHR, waist-to-hip ratio.

*p<0.050;**p<0.010;***p<0.001

[a]Model fit indices: GFI: 0.996, CFI: 0.972, and RMSEA: 0.049 in both assessments

REFERENCES

1. Boutelle KN, Jeffery RW, French SA: Predictors of vigorous exercise adoption and maintenance over four years in a community sample. The international journal of behavioral nutrition and physical activity 2004, 1(1):13.
2. Lahti J, Laaksonen M, Lahelma E, Rahkonen O: Changes in leisure-time physical activity after transition to retirement: a follow-up study. The international journal of behavioral nutrition and physical activity 2011, 8:36.
3. Panagiotakos DB, Pitsavos C, Lentzas Y, Skoumas Y, Papadimitriou L, Zeimbekis A, Stefanadis C: Determinants of physical inactivity among men and women from Greece: a 5-year follow-up of the ATTICA study. Annals of epidemiology 2008, 18(5):387-394.
4. Sallis JF, Haskell WL, Fortmann SP, Vranizan KM, Taylor CB, Solomon DS: Predictors of adoption and maintenance of physical activity in a community sample. Prev Med 1986, 15(4):331-341.
5. Weiss DR, O'Loughlin JL, Platt RW, Paradis G: Five-year predictors of physical activity decline among adults in low-income communities: a prospective study. The international journal of behavioral nutrition and physical activity 2007, 4:2.
6. Shaw BA, Liang J, Krause N, Gallant M, McGeever K: Age differences and social stratification in the long-term trajectories of leisure-time physical activity. The journals of gerontology Series B, Psychological sciences and social sciences 2010, 65(6):756-766.
7. Margolis R: Educational differences in healthy behavior changes and adherence among middle-aged Americans. J Health Soc Behav 2013, 54(3):353-368.
8. Hawkley LC, Thisted RA, Cacioppo JT: Loneliness predicts reduced physical activity: cross-sectional & longitudinal analyses. Health Psychology 2009, 28(3):354-363.

9. Sugiyama T, Giles-Corti B, Summers J, du Toit L, Leslie E, Owen N: Initiating and maintaining recreational walking: a longitudinal study on the influence of neighborhood green space. Prev Med 2013, 57(3):178-182.

10. Laaksonen M, Luoto R, Helakorpi S, Uutela A: Associations between health-related behaviors: a 7-year follow-up of adults. Prev Med 2002, 34(2):162-170.

11. Hull EE, Rofey DL, Robertson RJ, Nagle EF, Otto AD, Aaron DJ: Influence of marriage and parenthood on physical activity: a 2-year prospective analysis. Journal of Physical Activity and Health 2010, 7(5):577-583.

12. Picavet HS, Wendel-vos GC, Vreeken HL, Schuit AJ, Verschuren WM: How stable are physical activity habits among adults? The Doetinchem Cohort Study. Medicine and science in sports and exercise 2011, 43(1):74-79.

13. Sallis JF, Hovell MF, Hofstetter CR: Predictors of adoption and maintenance of vigorous physical activity in men and women. Prev Med 1992, 21(2):237-251.

14. Schmitz K, French SA, Jeffery RW: Correlates of changes in leisure time physical activity over 2 years: the Healthy Worker Project. Prev Med 1997, 26(4):570-579.

15. Brown WJ, Heesch KC, Miller YD: Life events and changing physical activity patterns in women at different life stages. Annals of behavioral medicine : a publication of the Society of Behavioral Medicine 2009, 37(3):294-305.

16. Chung S, Domino ME, Stearns SC, Popkin BM: Retirement and physical activity: analyses by occupation and wealth. Am J Prev Med 2009, 36(5):422-428.

17. Hamer M, Kivimaki M, Steptoe A: Longitudinal patterns in physical activity and sedentary behaviour from mid-life to early old age: a substudy of the Whitehall II cohort. Journal of epidemiology and community health 2012, 66(12):1110-1115.

18. Ranchod YK, Diez Roux AV, Evenson KR, Sánchez BN, Moore K: Longitudinal associations between neighborhood recreational facilities and change in recreational

physical activity in the multi-ethnic study of atherosclerosis, 2000-2007. American journal of epidemiology 2014, 179(3):335-343.

19. Dawson J, Hillsdon M, Boller I, Foster C: Perceived barriers to walking in the neighbourhood environment and change in physical activity levels over 12 months. British journal of sports medicine 2007, 41(9):562-568.

20. Shaw BA, Spokane LS: Examining the association between education level and physical activity changes during early old age. J Aging Health 2008, 20(7):767-787.

21. Zimmermann E, Ekholm O, Grønbaek M, Curtis T: Predictors of changes in physical activity in a prospective cohort study of the Danish adult population. Scandinavian journal of public health 2008, 36(3):235-241.

22. Cornelio CI, García M, Schiaffino A, Borrès JM, Nieto FJ, Fernández E; CHIS.FU Study Group. Changes in leisure time and occupational physical activity over 8 years: the Cornelle Health Interview Survey Follow-Up Study. Journal of epidemiology and community health 2008, 62(3):239-244.

23. O'Dougherty M, Hearst MO, Arikawa AY, Stovitz SD, Kurzer MS, Schmitz KH: Young women's physical activity from one year to the next: What changes? What stays the same? Translational behavioral medicine 2012, 2(2):129-136.

24. Stephan Y, Boiche J, Trouilloud D, Deroche T, Sarrazin P: The relation between risk perceptions and physical activity among older adults: a prospective study. Psychology & health 2011, 26(7):887-897.

25. Borodulin K, Mäkinen TE, Leino-Arjas P, Tammelin TH, Heliovaara M, Martelin T, Kestila L, Prattala R: Leisure time physical activity in a 22-year follow-up among Finnish adults. The international journal of behavioral nutrition and physical activity 2012, 9:121.

26. Eaton CB, Reynes J, Assaf AR, Feldman H, Lasater T, Carleton RA: Predicting physical

activity change in men and women in two New England communities. Am J Prev Med 1993, 9(4):209-219.

27. Macera CA, Croft JB, Brown DR, Ferguson JE, Lane MJ: Predictors of adopting leisure-time physical activity among a biracial community cohort. American journal of epidemiology 1995, 142(6):629-635.

28. Droomers M, Schrijvers CT, Mackenbach JP: Educational level and decreases in leisure time physical activity: predictors from the longitudinal GLOBE study. Journal of epidemiology and community health 2001, 55(8):562-568.

29. Boone-Heinonen J, Diez Roux AV, Kiefe CI, Lewis CE, Guilkey DK, Gordon-Larsen P: Neighborhood socioeconomic status predictors of physical activity through young to middle adulthood: the CARDIA study. Social science & medicine (1982) 2011, 72(5):641-649.

30. Pinto Pereira SM, Power C: Early adulthood determinants of mid-life leisure-time physical inactivity stability and change: Findings from a prospective birth cohort. Journal of science and medicine in sport 2018, 21(7):720-726.

31. Kaplan GA, Lazarus NB, Cohen RD, Leu DJ: Psychosocial factors in the natural history of physical activity. Am J Prev Med 1991, 7(1):12-17.

32. Sun H, Vamos CA, Flory SSB, DeBate R, Thompson EL, Bleck J: Correlates of long-term physical activity adherence in women. Journal of sport and health science 2017, 6(4):434-442.

33. Klesges RC, Stein RJ, Hultquist CM, Eck LH: Relationships among smoking status, body composition, energy intake, and physical activity in adult males: a longitudinal analysis. Journal of substance abuse 1992, 4(1):47-56.

34. Dishman RK, Vandenberg RJ, Motl RW, Nigg CR: Using constructs of the transtheoretical model to predict classes of change in regular physical activity: a multi-

ethnic longitudinal cohort study. Annals of behavioral medicine : a publication of the Society of Behavioral Medicine 2010, 40(2):150-163.

35. Titze S, Stronegger W, Owen N: Prospective study of individual, social, and environmental predictors of physical activity: women's leisure running. Psychology of Sport and Exercise 2005, 6(3):363-376.

36. Beenackers MA, Foster S, Kamphuis CB, Titze S, Divitini M, Knuiman M, van Lenthe FJ, Giles-Corti B: Taking up cycling after residential relocation: built environment factors. Am J Prev Med 2012, 42(6):610-615.

37. Courneya KS, Plotnikoff RC, Hotz SB, Birkett NJ: Predicting exercise stage transitions over two consecutive 6-month periods: a test of the theory of planned behaviour in a population-based sample. British journal of health psychology 2001, 6(Pt 2):135-150.

38. Fuchs R: Causal models of physical exercise participation: testing the predictive power of the construct “pressure to change”. Journal of Applied Social Psychology 1996, 26(21):1931-1960.

39. Julien D, Gauvin L, Richard L, Kestens Y, Payette H: Longitudinal associations between walking frequency and depressive symptoms in older adults: results from the VoisiNuAge study. Journal of the American Geriatrics Society 2013, 61(12):2072-2078.

40. Teychenne M, Abbott G, Lamb KE, Rosenbaum S, Ball K: Is the link between movement and mental health a two-way street? Prospective associations between physical activity, sedentary behaviour and depressive symptoms among women living in socioeconomically disadvantaged neighbourhoods. Prev Med 2017, 102:72-78.

41. Neutel CI, Campbell N; Canadian Hypertension Society. Changes in lifestyle after hypertension diagnosis in Canada. The Canadian journal of cardiology 2008, 24(3):199-204.

42. Newsom JT, Huguet N, McCarthy MJ, Ramage-Morin P, Kaplan MS, Bernier J,

McFarland BH, Oderkirk J: Health behavior change following chronic illness in middle and later life. The journals of gerontology Series B, Psychological sciences and social sciences 2012, 67(3):279-288.

43. Newson JT, Huguet N, Ramage-Morin PL, McCarthy MJ, Bernier J, Kaplan MS, McFarland BH: Health behaviour changes after diagnosis of chronic illness among Canadians aged 50 or older. Health reports 2012, 23(4):49-53.

44. van Gool CH, Kempen GI, Penninx BW, Deeg DJ, van Eijk JT: Chronic disease and lifestyle transitions: results from the Longitudinal Aging Study Amsterdam. J Aging Health 2007, 19(3):416-438.

45. Stults-Kolehmainen MA, Sinha R: The effects of stress on physical activity and exercise. Sports medicine (Auckland, NZ) 2014, 44(1):81-121.

46. Madsen IEH, Nyberg ST, Magnusson Hanson LL, Ferrie JE, Ahola K, Alfredsson L, Batty GD, Bjorner JB, Borritz M, Burr H, Chastang JF, de Graaf R, Dragano N, Hamer M, Jokela M, Knutsson A, Koskenvuo M, Koskinen A, Leineweber C, Niedhammer I, Nielsen ML, Nordin M, Oksanen T, Pejtersen JH, Pentti J, Plaisier I, Salo P, Singh-Manoux A, Suominen S, Ten Have M, Theorell T, Toppinen-Tanner S, Vahtera J, Väänänen A, Westerholm PJM, Westerlund H, Fransson EI, Heikkilä K, Virtanen M, Rugulies R, Kivimäki M; IPD-Work Consortium. Job strain as a risk factor for clinical depression: systematic review and meta-analysis with additional individual participant data. Psychological medicine 2017, 47(8):1342-1356.

47. Wardle J, Chida Y, Gibson EL, Whitaker KL, Steptoe A: Stress and adiposity: a meta-analysis of longitudinal studies. Obesity 2011, 19(4):771-778.

48. Booth J, Connelly L, Lawrence M, Chalmers C, Joice S, Becker C, Dougall N: Evidence of perceived psychosocial stress as a risk factor for stroke in adults: a meta-analysis. BMC neurology 2015, 15:233.

49. Liu MY, Li N, Li WA, Khan H: Association between psychosocial stress and hypertension: a systematic review and meta-analysis. Neurological research 2017, 39(6):573-580.

50. Milner A, Witt K, Spittal MJ, Bismark M, Graham M, LaMontagne AD: The relationship between working conditions and self-rated health among medical doctors: evidence from seven waves of the Medicine In Australia Balancing Employment and Life (Mabel) survey. BMC health services research 2017, 17(1):609.

51. Aronsson V, Toivanen S, Leineweber C, Nyberg A: Can a poor psychosocial work environment and insufficient organizational resources explain the higher risk of ill-health and sickness absence in human service occupations? Evidence from a Swedish national cohort. Scandinavian journal of public health 2019;47(3):310-317.

52. Magnusson Hanson LL, Westerlund H, Chungkham HS, Vahtera J, Rod NH, Alexanderson K, Goldberg M, Kivimaki M, Stenholm S, Platts LG *et al*: Job strain and loss of healthy life years between ages 50 and 75 by sex and occupational position: analyses of 64 934 individuals from four prospective cohort studies. Occupational and environmental medicine 2018, 75(7):486-493.

53. Burr H, Hasselhorn HM, Kersten N, Pohrt A, Rugulies R: Does age modify the association between psychosocial factors at work and deterioration of self-rated health? Scandinavian journal of work, environment & health 2017, 43(5):465-474.

54. Svedberg P, Bardage C, Sandin S, Pedersen NL: A prospective study of health, life-style and psychosocial predictors of self-rated health. Eur J Epidemiol 2006, 21(10):767-776.

55. Farmer MM, Ferraro KF: Distress and perceived health: mechanisms of health decline. J Health Soc Behav 1997, 38(3):298-311.

56. Shields M, Shooshtari S: Determinants of self-perceived health. Health reports 2001, 13(1):35-52.

57. McHugh JE, Lawlor BA: Executive functioning independently predicts self-rated health and improvement in self-rated health over time among community-dwelling older adults. Aging & mental health 2016, 20(4):415-422.

58. Hitsman B, Papandonatos GD, McChargue DE, DeMott A, Herrera MJ, Spring B, Borrelli B, Niaura R: Past major depression and smoking cessation outcome: a systematic review and meta-analysis update. Addiction (Abingdon, England) 2013, 108(2):294-306.

59. Barlinn K, Kepplinger J, Puetz V, Illigens BM, Bodechtel U, Siepmann T: Exploring the risk-factor association between depression and incident stroke: a systematic review and meta-analysis. Neuropsychiatric disease and treatment 2015, 11:1-14.

60. Sun H-L, Dong X-X, Cong Y-J, Gan Y, Deng J, Cao S-Y, Lu Z-X: Depression and the risk of breast cancer: a meta-analysis of cohort studies. Asian Pac J Cancer Prev. 2015;16(8):3233-3239.

61. Wagner DC, Short JL: Longitudinal predictors of self-rated health and mortality in older adults. Preventing chronic disease 2014, 11:E93.

62. Ayyagari P, Ullrich F, Malmstrom TK, Andresen EM, Schootman M, Miller JP, Miller DK, Wolinsky FD: Self-rated health trajectories in the African American health cohort. PloS one 2012, 7(12):e53278.

63. Chen H, Cohen P, Kasen S: Cohort differences in self-rated health: evidence from a three-decade, community-based, longitudinal study of women. American journal of epidemiology 2007, 166(4):439-446.

64. Deng G, Yin L, Liu W, Liu X, Xiang Q, Qian Z, Ma J, Chen H, Wang Y, Hu B, Li W, Jiang Y; China Investigator team. Associations of anthropometric adiposity indexes with hypertension risk: A systematic review and meta-analysis including PURE-China. Medicine 2018, 97(48):e13262.

65. Liu X, Sun Q, Hou H, Zhu K, Wang Q, Liu H, Zhang Q, Ji L, Li D: The association between BMI and kidney cancer risk: An updated dose-response meta-analysis in accordance with PRISMA guideline. Medicine 2018, 97(44):e12860.

66. Ashwell M, Gunn P, Gibson S: Waist-to-height ratio is a better screening tool than waist circumference and BMI for adult cardiometabolic risk factors: systematic review and meta-analysis. Obesity reviews 2012, 13(3):275-286.

67. Simonsen MK, Hundrup YA, Grønbaek M, Heitmann BL: A prospective study of the association between weight changes and self-rated health. BMC women's health 2008, 8:13.

68. Bäckmand H, Kujala U, Sarna S, Kaprio J: Former athletes' health-related lifestyle behaviours and self-rated health in late adulthood. International journal of sports medicine 2010, 31(10):751-758.

69. West R, Evins AE, Benowitz NL, Russ C, McRae T, Lawrence D, St Aubin L, Krishen A, Maravic MC, Anthenelli RM: Factors associated with the efficacy of smoking cessation treatments and predictors of smoking abstinence in EAGLES. Addiction (Abingdon, England) 2018, 113(8):1507-1516.

70. Ayala-Bernal D, Probst-Hensch N, Rochat T, Bettschart R, Brandli O, Bridevaux PO, Burdet L, Frey M, Gerbase M, Pons M *et al*: Factors associated with cessation of smoking among Swiss adults between 1991 and 2011: results from the SAPALDIA cohort. Swiss medical weekly 2017, 147:w14502.

71. Berg CJ, Thomas JL, Guo H, An LC, Okuyemi KS, Collins TC, Ahluwalia JS: Predictors of smoking reduction among Blacks. Nicotine & tobacco research : official journal of the Society for Research on Nicotine and Tobacco 2010, 12(4):423-431.

72. Nørregaard J, Tønnesen P, Petersen L: Predictors and reasons for relapse in smoking cessation with nicotine and placebo patches. Prev Med 1993, 22(2):261-271.

73. Sugawara Y, Tsuji I, Mizoue T, Inoue M, Sawada N, Matsuo K, Ito H, Naito M, Nagata C, Kitamura Y, Sadakane A, Tanaka K, Tamakoshi A, Tsugane S, Shimazu T; Research Group for the Development and Evaluation of Cancer Prevention Strategies in Japan: Cigarette smoking and cervical cancer risk: an evaluation based on a systematic review and meta-analysis among Japanese women. Japanese journal of clinical oncology 2019, 49(1):77-86.

74. O'Keeffe LM, Taylor G, Huxley RR, Mitchell P, Woodward M, Peters SAE: Smoking as a risk factor for lung cancer in women and men: a systematic review and meta-analysis. BMJ open 2018, 8(10):e021611.

75. Castillo JJ, Dalia S, Shum H: Meta-analysis of the association between cigarette smoking and incidence of Hodgkin's Lymphoma. Journal of Clinical Oncology 2011, 29(29):3900-3906.

76. Aune D, Schlesinger S, Norat T, Riboli E: Tobacco smoking and the risk of heart failure: A systematic review and meta-analysis of prospective studies. European journal of preventive cardiology 2019, 26(3):279-288.

77. Willi C, Bodenmann P, Ghali WA, Faris PD, Cornuz J: Active smoking and the risk of type 2 diabetes: a systematic review and meta-analysis. Jama 2007, 298(22):2654-2664.

78. Taylor G, McNeill A, Girling A, Farley A, Lindson-Hawley N, Aveyard P: Change in mental health after smoking cessation: systematic review and meta-analysis. BMJ (Clinical research ed) 2014, 348:g1151.

79. Sargent-Cox K, Cherbuin N, Morris L, Butterworth P, Anstey KJ: The effect of health behavior change on self-rated health across the adult life course: a longitudinal cohort study. Prev Med 2014, 58:75-80.

80. Perlman F, Bobak M: Determinants of self rated health and mortality in Russia - are they the same? International journal for equity in health 2008, 7:19.

81. Holden L, Harris M, Hockey R, Ferrari A, Lee YY, Dobson AJ, Lee C: Predictors of change in depressive symptoms over time: Results from the Australian Longitudinal Study on Women's Health. Journal of affective disorders 2019, 245:771-778.

82. Hwang WC, Myers HF, Takeuchi DT: Psychosocial predictors of first-onset depression in Chinese Americans. Social psychiatry and psychiatric epidemiology 2000, 35(3):133-145.

83. Handley TE, Rich J, Lewin TJ, Kelly BJ: The predictors of depression in a longitudinal cohort of community dwelling rural adults in Australia. Social psychiatry and psychiatric epidemiology 2019, 54(2):171-180.

84. Misawa J, Kondo K: Social factors relating to depression among older people in Japan: analysis of longitudinal panel data from the AGES project. Aging & mental health 2019;23(10):1423-1432.

85. Carlson LE, Goodey E, Bennett MH, Taenzer P, Koopmans J: The addition of social support to a community-based large-group behavioral smoking cessation intervention: improved cessation rates and gender differences. Addictive behaviors 2002, 27(4):547-559.

86. Albertsen K, Borg V, Oldenburg B: A systematic review of the impact of work environment on smoking cessation, relapse and amount smoked. Prev Med 2006, 43(4):291-305.

87. Nides MA, Rakos RF, Gonzales D, Murray RP, Tashkin DP, Bjornson-Benson WM, Lindgren P, Connett JE: Predictors of initial smoking cessation and relapse through the first 2 years of the Lung Health Study. Journal of consulting and clinical psychology 1995, 63(1):60-69.

88. Jaén CR, Cummings KM, Zielezny M, O'Shea R: Patterns and predictors of smoking cessation among users of a telephone hotline. Public health reports (Washington, DC :

1974) 1993, 108(6):772-778.

89. Curry S, Thompson B, Sexton M, Omenn GS: Psychosocial predictors of outcome in a worksite smoking cessation program. Am J Prev Med 1989, 5(1):2-7.

90. Steinmetz-Wood M, Gagné T, Sylvestre MP, Frohlich K: Do social characteristics influence smoking uptake and cessation during young adulthood? International journal of public health 2018, 63(1):115-123.

91. Blok DJ, de Vlas SJ, van Empelen P, van Lenthe FJ: The role of smoking in social networks on smoking cessation and relapse among adults: A longitudinal study. Prev Med 2017, 99:105-110.

92. May S, West R, Hajek P, McEwen A, McRobbie H: Randomized controlled trial of a social support ('buddy') intervention for smoking cessation. Patient Educ Couns 2006, 64(1-3):235-241.

93. Ota A, Masue T, Yasuda N, Tsutsumi A, Mino Y, Ohara H, Ono Y: Psychosocial job characteristics and smoking cessation: a prospective cohort study using the Demand-Control-Support and Effort-Reward Imbalance job stress models. Nicotine & tobacco research : official journal of the Society for Research on Nicotine and Tobacco 2010, 12(3):287-293.

94. Sperber AD, Goren-Lerer M, Peleg A, Friger M: Smoking cessation support groups in Israel: a long-term follow-up. The Israel Medical Association journal : IMAJ 2000, 2(5):356-360.

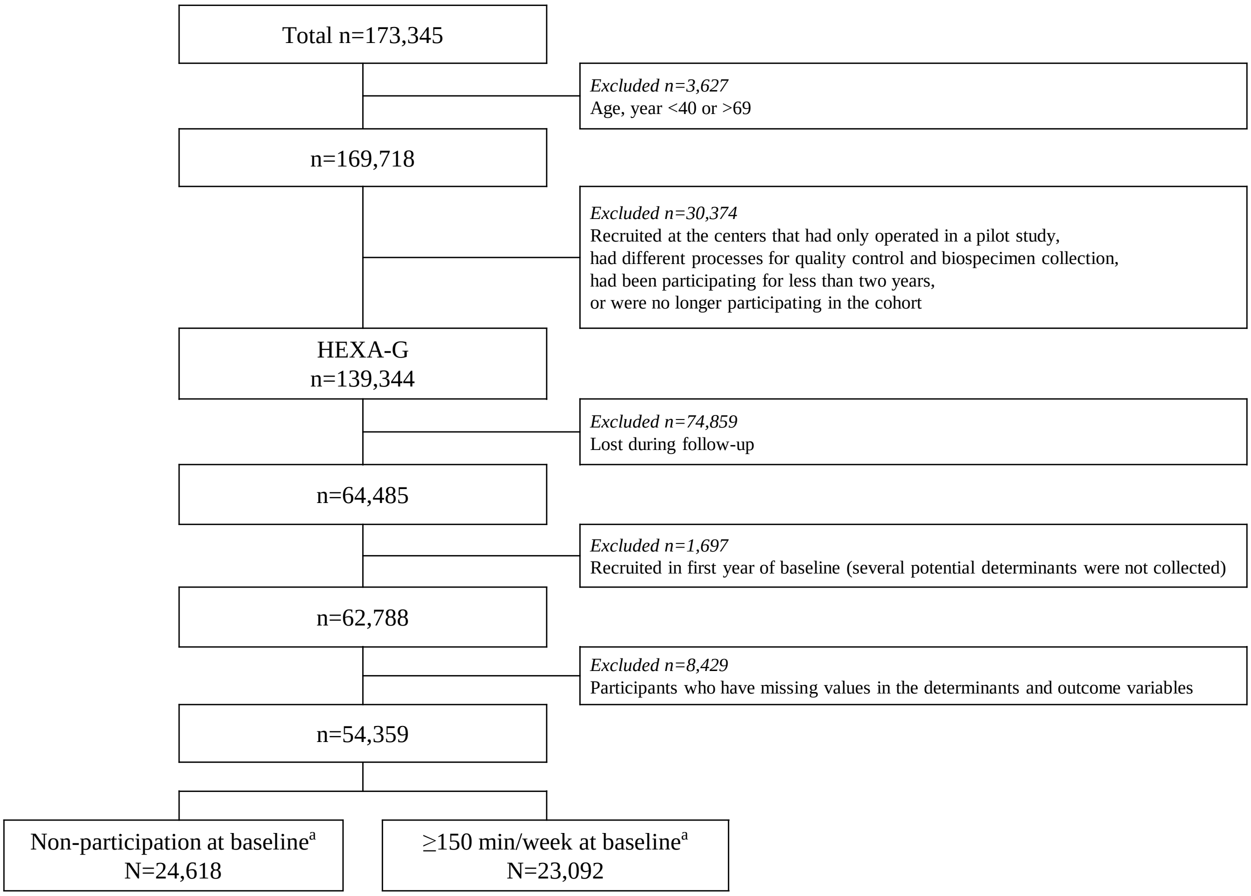

eFigure 1. Inclusion and exclusion criteria of study population

[a]Associations of determinants with initiation and maintenance of LTPA were evaluated in those who did not participate in physical activity at baseline and participate in 150 minutes or more per week of physical activity at baseline, respectively. LTPA, leisure time physical activity.

Overall leisure-time physical activity analysis (total population, N=54,359)

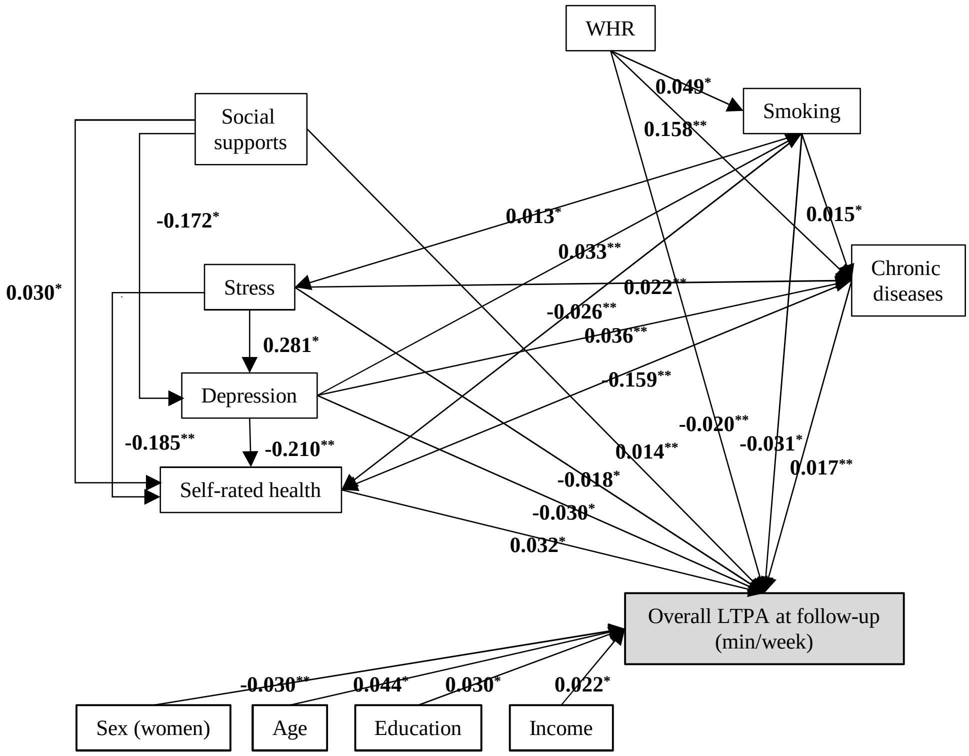

eFigure 2. Standardized coefficients of direct effect of potential determinants at baseline and the duration of overall leisure-time physical activity at follow-up

Only significant associations are presented
Model fit indices: GFI: 0.996, CFI: 0.972, and RMSEA: 0.049 in duration model
*p<0.05;**p<0.01;***p<0.001
LTPA: leisure-time physical activity; WHR, waist-to-hip ratio
